# Editorial: Evolution of Gene Regulatory Networks in Plant Development

**DOI:** 10.3389/fpls.2017.02126

**Published:** 2017-12-15

**Authors:** Federico Valverde, Andrew Groover, José M. Romero

**Affiliations:** ^1^Plant Development Unit, Institute for Plant Biochemistry and Photosynthesis, Consejo Superior de Investigaciones Científicas-Universidad de Sevilla, Seville, Spain; ^2^US Forest Service, Pacific Southwest Research Station, Davis, CA, United States; ^3^Department of Plant Biology, University of California, Davis, Davis, CA, United States; ^4^Departamento de Bioquímica Vegetal y Biología Molecular, Universidad de Sevilla, Seville, Spain

**Keywords:** gene regulatory networks, plant development, evolution, omics, molecular system biology

The mechanisms regulating developmental processes in plants are very sophisticated as a result of a continuous increase in complexity during evolution. Plants undergo a wide variety of morphological and developmental changes in their life time. The regulation of gene expression is a primary mechanisms of regulating development. Gene expression is controlled by regulators that, together with their regulatory interactions, integrate environmental cues and coordinate different developmental programmes (Kaufmann and Chen, 2017) through gene regulatory networks (GRNs). GRNs can be defined as a series of regulatory factors that interact with each other and with other regulators to control the levels of mRNA and proteins to specify temporal and spatial patterns (Davidson and Levin, 2005; Levine and Davidson, 2005), and often involve interactions with metabolic networks. New technological advances use the computational analysis of massive “omics” data to generate holistic views of complex biological processes. This Research Topic focuses on the evolution of genes, gene families and GRNs involved in plant development.

Plant growth and development is widely regulated by the circadian clock, which allows plants to anticipate light transitions. The GRN of the circadian clock generates 24-h rhythms influencing various aspects of plant biology including gene expression, metabolism, developmental programmes and transitions, such as flowering (Millar, 2016; Nohales and Kay, 2016). A significant number of genes are regulated by the circadian clock in photosynthetic organisms. It is estimated that in algae about 80–90% and in plants between 30 and 50% of their genes show daily rhythmic patterns (Covington et al., 2008; Zones et al., 2015). That means that despite the evolutionary distance between algae and plants, the expression patterns of many gene clusters showing daily rhythms are conserved (de los Reyes et al.; Serrano-Bueno et al., 2017). Analysis of gene-co-expression networks in plants and microalgae and the use of a new algorithm MBBH (Multiple Bidirectional Best Hits) has allowed the identification of orthologous genes from species evolutionarily very distant, and reveal a wide conservation of genes under daily rhythms in the green lineage (de los Reyes et al.). Photoperiodic sensing (the detection of day length) is crucial for plants to make important decisions during their life, such as the best time to flower. Photoperiod sensing is closely associated with photoreceptor signaling and the circadian clock, processes that are conserved during the evolution of the green lineage (Serrano-Bueno et al., 2017). Brambilla et al. describe the GRNs controlling photoperiodic flowering in long-day and short-day cereals showing their flexibility and how the photoperiodic gene networks have evolved in crops. They also discuss the fact that some regulators are not conserved in all lineages, and that several conserved elements of these GRNs are dedicated to novel functions.

Among many other developmental processes in plants, floral transition and the subsequent floral organ development are triggered by both internal and external cues and are coordinated by gene networks highly conserved in evolution (Romero-Campero et al., 2013). Floral organ identity is determined by a GRNs defined by MADS-box transcription factors, according to the ABCE model for floral development (Lucas-Reina et al., 2016). The *APETALA2* (*AP2*) gene participates in the determination of sepals and petals, belonging to the *AP2/ERF* family and being the only gene of the ABCE model that is not a MADS-box. A comprehensive analysis of the *AP2* genes in a series of spermatophytes indicates that these genes suffered from both negative and positive selection through evolution, first appearing in Gymnosperms and evolved by gene duplication in Angiosperms (Wang et al.). The formation of carpels is directed by the combined action of the C and E functions of the flower organogenesis model. However, carpel development requires a plethora of regulatory genes some of them related to auxin signaling. The *SHORT INTERNODES* (*SHI*), *STYLISH* (*STY*), and *SHI RELATED SEQUENCE* (*SRS*) gene family of zinc-finger transcription factors (*SHI/STY/SRS*) is involved in carpel formation in distantly related flowering plants as *Arabidopsis thaliana* and *Nicotiana benthamiana*, suggesting a common evolutionary origin of the GRN directing carpel development, and possibly the conservation of their targets (Gomariz-Fernández et al.). Ovule development, fruit shape, size and ripening are controlled by multiple factors. The OVATE family of proteins (OFP) is specific to plants and was first identified because of their influence on fruit shape and size in tomato, and constitute a large gene family in all plants studied. OFPs participate in many aspects of plant development and growth as ovule development, fruit shape, fruit ripening, vascular development or even DNA repair (Wang et al.), however further evidence is needed to understand their function. In the developmental programme of seeds, many plants generate a variety of structures related to seed protection and dispersion. Arils are structures, often fleshy, that can accumulate sugars and other substances that help dispersion. Due to the fact that plant model systems do not develop arils, little is known about their origin and the molecular GRN involved in its formation. The origin and the state of the art of aril development and evolution, as well as the molecular pathways known to date is covered by Silveira et al.

The MADS-box family of transcription factors is implicated in flower development, as indicated above, but also in many other developmental and growth programmes. A comprehensive analysis of the MADS-box family in radish (*Raphanus sativus*) genome indicates that 144 genes code for MADS-box proteins. These data in combination with the characterization of their differential expression pattern in diverse developmental stages and tissues by Next Generation Sequencing (NGS), provides a basic tool to study flowering and floral development in radish, an economically important root crop (Li et al.).

The form and symmetry of the flower in plants is extremely variable. Flower symmetry is controlled by a set of transcription factors including CYCLOIDEA (CYC) and DICHOTOMA (DICH), originally characterized in the eudicot *Antirrhinum majus* (Luo et al., 1999), that belong to the *TCP* gene family (*TEOSINTE BRANCHED* (*TB*), *CYCLOIDEA* (*CYC*), and *PROLIFFERATION CELL FACTOR* (*PCF*). It is considered that the success of *Asteraceae*, one of the largest family of vascular plants, is related to their head-like inflorescences, or *capitulum* (Broholm et al., 2014). By analysing the phylogeny of *CYC/TB1* genes together with their expression patterns in *Anacyclus* flowers, it has been shown that *CYC* paralogs play a largely conserved role in determining floral symmetry (Bello et al.). Although the flower structure of monocots differs significantly from dicots, the role of the genes involved in flower symmetry are evolutionarily conserved. Madrigal et al. performed a large-scale study of the *TCP* gene family and their pattern of expression in *Asparagales* and showed that in this monocot group the number of *CYC*-like genes is reduced in relation to eudicots, while the converse is observed for *PCF*-like and *CINCINNATA*-like genes. This analysis provides basic information for further functional studies on flower symmetry in monocots. Although TCP transcription factors were first characterized as floral symmetry regulators, they are also involved in different developmental processes affecting growth and plant architecture. TCPs connect growth responses (including interactions with plant hormones) to the changing environment as light quality, abiotic and biotic stress or the availability of nutrients (Danisman). TCP transcription factors are also involved in bud dormancy. The *Arabidopsis* BRANCHED1 (BRC1), a TCP transcription factor, is implicated within the bud in the control of bud dormancy (Aguilar-Martinez et al., 2007) and regulates different GRNs. The analysis of transcriptomic data has allowed the identification of four interconnected GRNs associated with bud dormancy in *A. thaliana* (Tarancón et al.). They share a significant proportion of genes, indicating that they act coordinately, possibly through hormonal control. Several genes related to carbon starvation response are present in these GRNs, and it has been shown that this syndrome precedes the transition to dormancy both in herbaceous and woody plants, suggesting that bud dormancy could be an ancient response to unfavorable environmental conditions (Tarancón et al.).

Plant hormones play a crucial role in interpreting environmental signals (detected by receptors) and induce developmental programmes through different interconnected pathways. Gibberellins (GAs) induce the degradation of DELLA proteins (named by the amino acid sequence D-E-L-L-A in their N-terminal region) allowing the integration of environmental signals by controlling over 100 transcription factors. By the generation of gene co-expression networks in different organisms, it has been proposed that DELLA proteins had a basic role in coordinating diverse developmental programmes during evolution of the green lineage. The ancient DELLA proteins present in bryophytes do not respond to GAs as in vascular plants. Thus, the recruitment of DELLAs in the gibberellin signaling pathway might have increased their importance in coordinating different processes (Briones-Moreno et al.). Sex determination in monoecious plants is controlled, among other factors, by plant hormones like ethylene and GAs. In cucumber (*Cucumis sativus* L.), a monoecious plant, GAs are involved in the formation of the male flower. Transcriptomic analysis of shoot apices in GA-treated and control cucumber plants have provided evidences that GA regulation of sex determination takes place through both, ethylene –dependent and ethylene-independent pathways (Zhang et al.).

The function and components of many GRNs are conserved in evolution based in the innovation, amplification and divergence theory (Bergthorsson et al., 2007; Romero-Campero et al., 2013). Genome-wide analysis (GWA) is a potent tool to study gene families and determine how the different members evolved. In this context, studies of the JmjC domain-containing proteins, involved in histone demethylation, provide insights into the evolution of this group of proteins after genome duplication in soybean (Han et al.), which includes 48 putative *JmjC* genes. Also, the components of the Calcium Dependent Protein Kinases/Snf-1(CDPK-SnRK) superfamily from *Brassica rapa*, whose genome was modified by a whole-genome triplication (Cheng et al., 2014), were determined (Wu et al.). CDPK/SnRK proteins have important roles in multiple processes including the response to stress, sugar signaling and seed germination, among others (Kudla et al., 2010). Evolutionary studies comparing the CDPK/SnRK family from different plants representing the major clades provided insights into the evolutionary history of this protein family in plants (Wu et al.). The characterization of the *KNOTTED HOMEOBOX* (*KNOX*) coding genes from different *Cactaceae* and the determination of their expression pattern in the cambial zone provided clues to understand wood formation in this group of plants. This, together with phylogenetic analysis enlightens the evolutionary history of the *KNOX* gene family (Reyes-Rivera et al.). Comparative analysis of functions in different plant systems has allowed the identification of similarities and differences among species that caused the establishment of similar functions. PPR (Pentatricopeptide Repeat) containing proteins constitutes a family of proteins involved in the establishment of organelle RNA levels and participate in organelle biogenesis (Barkan and Small, 2014). Comparative analysis of CRP1 (Chloroplast RNA Processing 1), belonging to the PPR protein family, from *Arabidopsis* (AtCRP1) and maize (ZmCRP1) showed the conservation of the functionality of these genes between monocots and dicots (Ferrari et al.).

This Research Topic is intended to deepen our understanding of the evolution of GRNs underlying different plant developmental pathways and processes. The papers herein describing and comparing GRNs associated with diverse processes including photoperiod, circadian clock, hormone regulation, pattern formation, phase-transitions, organ development, etc. illustrate how the regulatory networks of plants are organized and evolved. We hope that the work presented in this Research Topic will support the growing international efforts in molecular systems biology that are providing exciting new insights into developmental processes in plants.

## Author contributions

All authors listed, have made substantial, direct and intellectual contribution to the work, and approved it for publication.

### Conflict of interest statement

The authors declare that the research was conducted in the absence of any commercial or financial relationships that could be construed as a potential conflict of interest.
